# Shape Effects of Cylindrical versus Spherical Unimolecular Polymer Nanomaterials on in Vitro and in Vivo Behaviors

**DOI:** 10.34133/2019/2391486

**Published:** 2019-04-24

**Authors:** Zhengkui Zhang, Changren Liu, Cheng Li, Wei Wu, Xiqun Jiang

**Affiliations:** Department of Polymer Science & Engineering, College of Chemistry & Chemical Engineering, Nanjing University, Nanjing 210023, China

## Abstract

To date, how the shape of nanomaterials influences their biological properties is poorly understood, due to the insufficient controllability of current preparative methods, especially in the shape and size of nanomaterials. In this paper, we achieved the precise syntheses of nanoscale unimolecular cylindrical polymer brushes (CPBs) and spherical polymer nanoparticles (SPNPs) with the same volume and surface chemistry, which ensured that shape was essentially the only variable when their biological performance was compared. Accurate shape effects were obtained. Impressively, the CPBs had remarkable advantage in tissue penetration over the SPNPs. The CPBs also exhibited higher cellular uptake and rapider body clearance than the SPNPs, whereas the SPNPs had longer blood circulation time, rapider tumor vascular extravasation, and higher tumor accumulation than the CPBs. Additionally, this work also provided a controllable synthesis strategy for nanoscale unimolecular SPNPs by integrating 21 CPBs to a *β*-cyclodextrin core, whose diameter in dry state could be up to 45 nm.

## 1. Introduction

Although nanomedicines for tumor therapy are encountering dramatic challenges in clinical applications due to their unsatisfactory treatment effects [1–5], they remain very promising since a large variety of nanomaterials with diverse structures and functions can be used as drug carriers and provide great potentials for the development of nanomedicines [6–13]. The limited treatment effects of nanomedicines are mainly caused by the drug resistance, physical barriers, tumor heterogeneity, and metastasis [1, 6]. To overcome these obstacles, we need to understand definitely the effects of the physicochemical properties of nanomaterials on their biological properties and design more efficient drug delivery systems [2, 14, 15].

The effects of the size and surface chemistry of different types of nanomaterials on their in vitro and in vivo behaviors have been studied by many research groups, which involves polymer micelles [16], polymer nanoparticles (NPs) [17, 18], dendrimers [19, 20], silica NPs [21], gold NPs [22, 23], graphene oxide [24], etc. However, to date, only a small number of studies have involved the shape effects of nanomaterials on their biological performance, because it is very difficult to control the nanomaterial shape at will, especially for polymer nanomaterials. Discher and coworkers prepared cylindrical polymer micelles (known as filomicelles) by the self-assembly of block copolymers and demonstrated that the filomicelles with length larger than 8 *μ*m had a blood retention time up to one week after intravenous injection, which was much longer than those of short filomicelles and spherical counterparts [25]. Later, an opposite length-dependent behavior was observed on cylindrical polymer brushes (CPBs). CPBs, also termed molecular polymer brushes (MPBs), molecular brushes, bottlebrushes, or densely grafted polymers in literatures, have typical one-dimensional morphological structures [26, 27]. Caruso et al. studied the biological performance of the CPBs with lengths ranged from 35 nm to 1.2 *μ*m (the CPBs with 35 nm length were assumed to be spherical), showing that the blood retention time of the CPBs decreased with the increase of brush length [28]. The distinct shape-dependency exhibited by the filomicelles and CPBs may be associated with their different size range and different chemical and morphological structures. It is worth noting that, in these shape effect studies, the compared cylindrical and spherical materials have considerably different volumes. That is to say, the causes of the differences in biological properties are not only the shape but also the volume. For obtaining the real shape effects, it is important to make shape be the only variable with other parameters including volume and surface chemistry similar. Polystyrene spheres and rods with the same volume were prepared in micro- and nanoscales by using film-stretching method, in which the polystyrene rods were fabricated by stretching polystyrene spheres above their glass-transition temperature (T_g_) followed by freezing the new shape below the T_g_ [29]. It was demonstrated that the polystyrene rods had reduced macrophage internalization, prolonged circulation time, and enhanced targeting to lungs compared to the spherical counterparts [30–33]. However, this physical preparation method demands that the polymer starting materials have proper T_g_, which strongly reduces the availability of this method. Furthermore, the polystyrene micro/nanomaterials have hydrophobic nature and limited stability due to the uncrosslinked structure, and their functionalizations can generally only be achieved by noncovalent approaches, for example, surface coating. All these greatly limit their application in biomedical field.

In this work, we present the precise syntheses of CPBs and unimolecular spherical polymer nanoparticles (SPNPs) with the same volume and surface chemistry. Their polydispersity indexes (Ð) are very close to 1.1 as measured by gel permeation chromatography (GPC). Both the two types of polymers have desirable solubility in water and abundant reactive groups for functionalizations. The CPBs have a flexible wormlike structure with a large aspect ratio of ~12, which is significantly different from the rigid structure of the reported polystyrene rods that have relatively small aspect ratios [30–32]. The in vitro and in vivo properties of the CPBs and SPNPs, including cellular uptake in different cell lines, phagocytosis by RAW264.7 cells, penetration in three-dimensional (3D) multicellular spheroids (MCs), biodistribution, and extravasation behaviors from tumor vessels, were systematically studied and compared.

## 2. Results

### 2.1. Syntheses and Characterizations of the CPBs and SPNPs

**Scheme 1:**
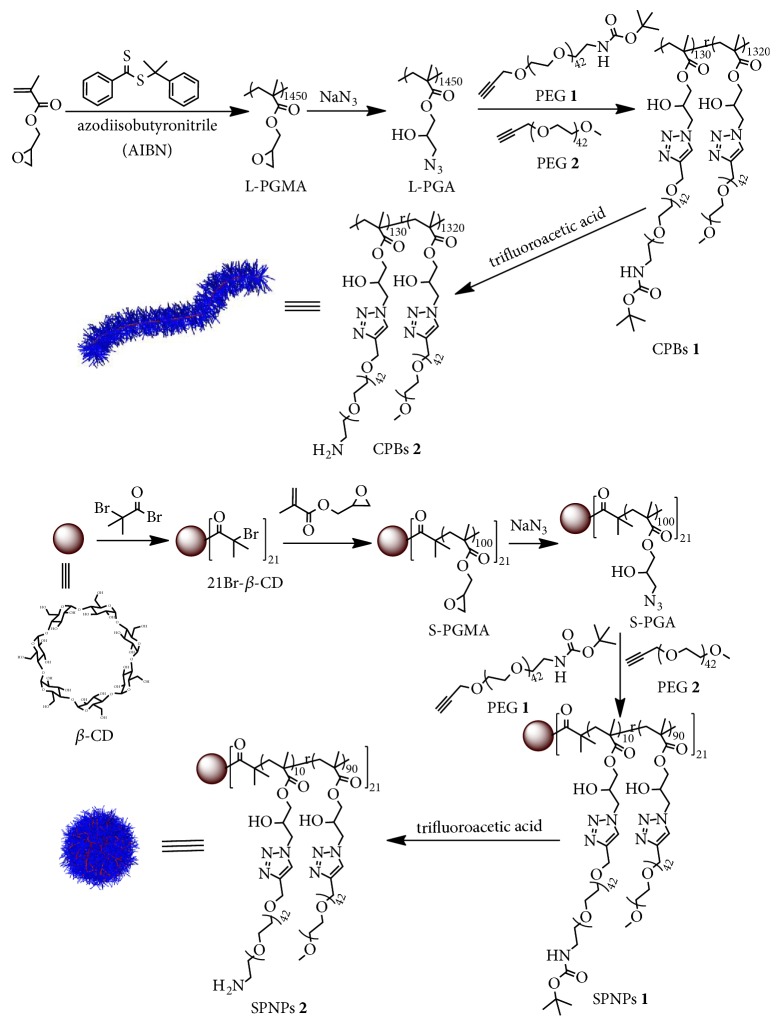
Synthetic routes of the CPBs and SPNPs.

The synthetic routes of the CPBs and SPNPs are summarized in Scheme 1. The backbone of the CPBs is the azido functionalized polymer L-PGA (Scheme 1), which was derived from linear poly(glycidyl methacrylate) (L-PGMA) by postmodification with sodium azide. Briefly, the L-PGMA was synthesized by reversible addition-fragmentation chain-transfer (RAFT) polymerization with cumyldithiobenzoate as a RAFT agent. Thereafter, the epoxy side groups in L-PGMA were converted into azido groups to give L-PGA for densely grafting poly(ethylene glycol) (PEG) chains by Cu(I)-catalyzed alkyne-azide 1,3-dipolar cycloaddition (CuAAC). In this work, we used two types of PEG as the side chains to prepare the CPBs (Scheme 1). The first one named PEG** 1** is a heterobifunctional PEG bearing an alkynyl group at one end and a* tert*-butyloxycarbonyl (Boc)-protected amino group at the other end. The second one named PEG** 2** is PEG monomethyl ether monopropargyl ether. CPBs** 1** were then obtained by the CuAAC of the azido side groups with a mixture of PEG** 1**/PEG** 2** (molar ratio = 1:10). The Boc-protected amino groups in CPBs** 1** were used in the fluorescent and radioactive labeling of the CPBs after deprotection. To ensure the same surface chemistry and volume of the SPNPs as the CPBs, we prepared SPNPs** 1** by a three-step synthesis, in which a 21-arm star-shaped poly(glycidyl methacrylate) (S-PGMA) was first synthesized by atom transfer radical polymerization (ATRP) from a *β*-cyclodextrin (*β*-CD) core with 21 initiating sites (21Br-*β*-CD, Scheme 1, its ^1^H NMR spectrum is shown in [Supplementary-material supplementary-material-1]), and then the epoxy groups in S-PGMA were converted into azido groups by the reaction with sodium azide to give S-PGA, and finally the PEG** 1**/PEG** 2** (feeding molar ratio = 1:10) chains were grafted to the arms of S-PGA by CuAAC. By this synthetic strategy, a molecule of SPNPs** 1** can be looked as 21 polymer brushes diverging from a *β*-CD core. Therefore, the chemical structures of CPBs** 1** and SPNPs** 1** are very similar. In the preparation of SPNPs** 1**, we carefully controlled the polymerization degree of the arms to ensure the same volume between CPBs** 1** and SPNPs** 1**. After the cleavage of the Boc protecting groups, CPBs** 2** and SPNPs** 2** were obtained with amino groups on periphery (Scheme 1). Both CPBs** 2** and SPNPs** 2** are soluble in water, dimethylformamide (DMF), and dimethyl sulfoxide (DMSO).

**Figure 1:**
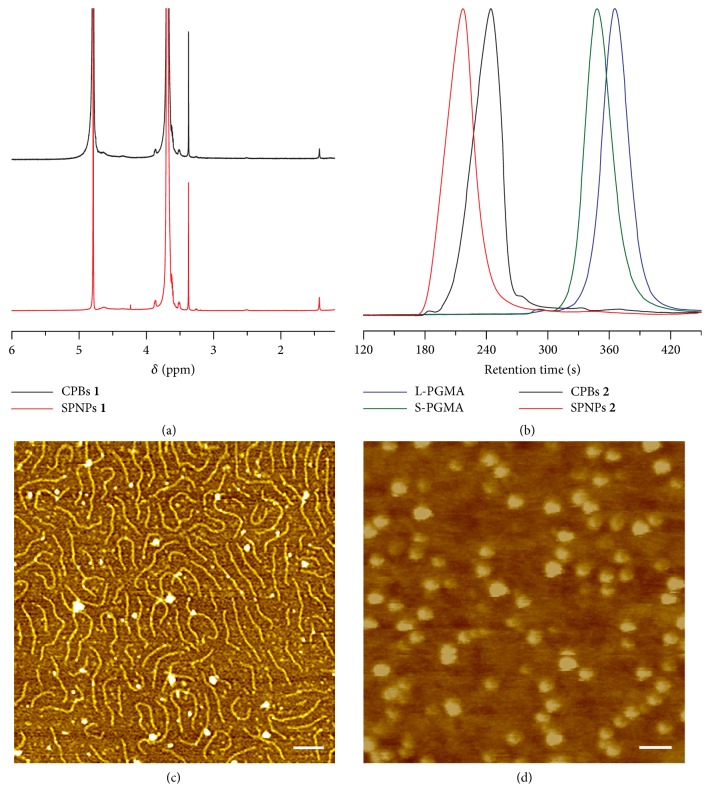
*Characterizations of the polymers*. (a) ^1^H NMR spectra of CPBs** 1** and SPNPs** 1** in D_2_O. (b) GPC curves of L-PGMA, S-PGMA, CPBs** 2**, and SPNPs** 2** with DMF as mobile phase. Typical AFM height images of CPBs** 2** (c) and SPNPs** 2** (d) adsorbed on mica from dilute water solutions. Scale bars = 100 nm.

The chemical structures of CPBs** 1** and SPNPs** 1** were characterized by ^1^H NMR (Figures 1(a) and [Supplementary-material supplementary-material-1]). It can be seen that the spectra of CPBs** 1** and SPNPs** 1** are almost the same since the only difference between their chemical structures is the *β*-CD core of SPNPs** 1** that contains very small proportion of protons. In the ^1^H NMR spectra of CPBs** 1** and SPNPs** 1**, the signals at 1.43 ppm are from the Boc protecting groups and the signals from 3.30 to 3.90 ppm are attributable to the PEG side chains. In either CPBs** 1** or SPNPs** 1**, the molar ratio of the side chains derived, respectively, from PEG** 1** and PEG** 2** is close to their feeding ratio (1:10), as determined by comparing the integral intensities of the signals between the Boc groups in PEG** 1** moieties (1.43 ppm) and the methyls in PEG** 2** moieties (3.37 ppm) ([Supplementary-material supplementary-material-1]). The ^1^H NMR spectra of CPBs** 2** and SPNPs** 2** show the disappearance of the signals from the Boc protecting groups, confirming the complete deprotection ([Supplementary-material supplementary-material-1]).

The molecular weights and distributions of L-PGMA and S-PGMA determine the sizes and distributions of the CPBs and SPNPs, respectively. As measured by GPC with DMF mobile phase (Figure 1(b)), both L-PGMA and S-PGMA exhibit unimodal molecular weight distributions. The number average molecular weight of L-PGMA is ~217,100 with* Ð* value of 1.09, and the number average molecular weight of S-PGMA is ~300,000 with* Ð* value of 1.10. Although the molecular weight of S-PGMA may be misestimated to some extent since the GPC measurements are calibrated with linear polystyrene standards, the GPC data demonstrate that both L-PGMA and S-PGMA have narrowly distributed molecular weights, which are attributable to the good controllability of the RAFT and ATRP polymerization methods.

We also examined the molecular weight distributions of CPBs** 2** and SPNPs** 2** by GPC by using DMF as mobile phase (Figure 1(b)), though their molecular weights could not be precisely determined with linear polymers as standards. Unimodal distributions are also observed in the GPC curves of CPBs** 2** and SPNPs** 2**, and* Ð *values of CPBs** 2** and SPNPs** 2** are calculated to be 1.13 and 1.11, respectively, definitely confirming their narrowly distributed size.

We further studied the morphological structures of CPBs** 2** and SPNPs** 2** by atomic force microscopy (AFM, Figures 1(c) and 1(d), 3D AFM images and cross-sections of CPBs** 2** and SPNPs** 2** can be found in [Supplementary-material supplementary-material-1]). It can be seen that CPBs** 2** have a wormlike structure with a narrowly distributed size (their length is ~203 nm and diameter is ~17 nm) and SPNPs** 2** exhibit a typical spherical structure with a narrowly distributed diameter (~45 nm). Based on their sizes and geometries, the volumes of CPBs** 2** and SPNPs** 2** can be calculated to be about 4.61 × 10^4^ nm^3^ and 4.77 × 10^4^ nm^3^, respectively, and are almost the same. It can also be seen that the wormlike structure of CPBs** 2** is very flexible, which results in much variability in the spatial arrangement of CPBs** 2** molecules. We have also tried to observe CPBs** 2** and SPNPs** 2 **by using transmission electron microscopy (TEM). However, we failed to see CPBs** 2** because their thickness and chain density are not sufficient to create enough electron density contrast for TEM analysis. We observed SPNPs** 2** clearly by TEM, showing a spherical structure with a uniform size and an average diameter of ~43 nm ([Supplementary-material supplementary-material-1]) and thus confirming the AFM results. The same volume and surface chemistry of CPBs** 2** and SPNPs** 2** together with their narrowly distributed sizes ensure the desirable accuracy of the shape effect studies.

### 2.2. In Vitro Cytotoxicity and Cellular Uptake

To preliminarily assess the biosafety of CPBs** 2** and SPNPs** 2**, we measured their in vitro cytotoxicities against human alveolar adenocarcinoma cells (A549), human neuroblastoma SH-SY5Y cells and human umbilical vein endothelial cells (HUVECs) by 3-(4,5-dimethylthiazol-2-yl)-2,5-diphenyltetrazolium bromide (MTT) assay after 24 h incubation with CPBs** 2** and SPNPs** 2** at different dosages, respectively ([Supplementary-material supplementary-material-1]). As can be seen, for each of the three cell lines, no significant cytotoxicity is observed for either CPBs** 2** or SPNPs** 2** at all the test concentrations, suggesting their good cytocompatibility.

**Figure 2:**
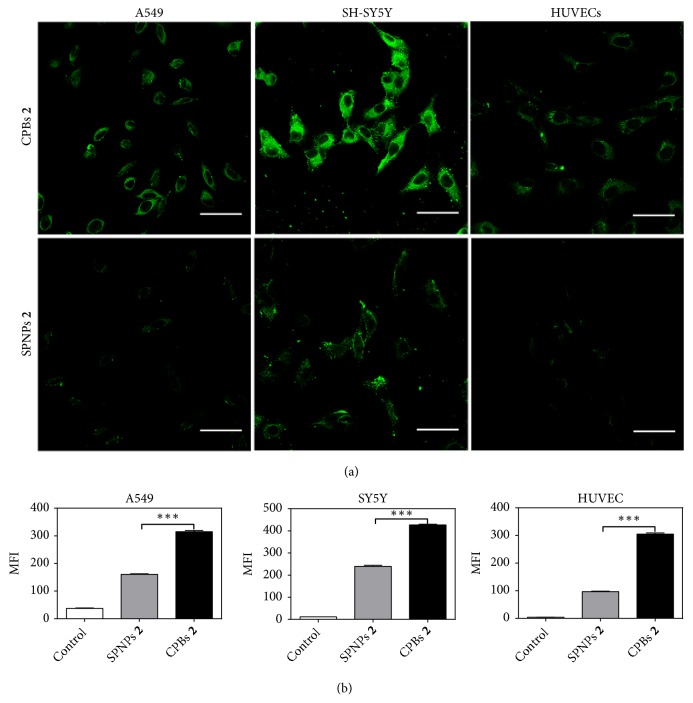
*Cellular uptakes of CPBs* ***2*** *and SPNPs* ***2***. (a) CLSM images of A549, SH-SY5Y, and HUVEC cells after 4 h incubation with the FITC-labeled CPBs** 2** and SPNPs** 2** at 37°C, respectively. Scale bars = 20 *μ*m. (b) Mean fluorescence intensity (MFI) in A549, SH-SY5Y, and HUVEC cells measured by flow cytometry after 4 h incubation with the FITC-labeled CPBs** 2** and SPNPs** 2** at 37°C, respectively. Data as mean values ± SD (n = 3). *∗∗∗P* < 0.001 (CPBs** 2** versus SPNPs** 2**).

The cellular uptakes of CPBs** 2** and SPNPs** 2** in A549 cells were compared qualitatively and quantitatively by confocal laser scanning microscopy (CLSM) and flow cytometry, respectively. CPBs** 2** and SPNPs** 2** were labeled with fluorescein isothiocyanate (FITC) through the reaction of the amino groups in CPBs** 2** and SPNPs** 2** with the isothiocyanate group in the dye. The fluorescence spectra and the plots of fluorescence intensity versus concentration of the labeled CPBs** 2** and SPNPs** 2** are shown in [Supplementary-material supplementary-material-1], revealing that the labeled CPBs** 2** and SPNPs** 2** have comparable fluorescence intensity at the same concentration.  Figure 2(a) shows the typical CLSM images of A549 cells after incubation with the FITC-labeled CPBs** 2** and SPNPs** 2** at 37°C for 4 h, respectively. As can be seen, both the labeled CPBs** 2** and SPNPs** 2** can be internalized by the cells and are distributed outside the nuclei in a punctuate pattern, suggesting that the cellular uptakes of the two materials are most likely to be conducted by endocytosis. Furthermore, the fluorescence intensity from the CPBs** 2** in the cells is significantly higher than that from the SPNPs** 2**, indicating that the cellular uptake of CPBs** 2** is significantly higher than that of SPNPs** 2**. The quantitative analyses of the cellular uptakes of the labeled CPBs** 2** and SPNPs** 2** by flow cytometry also show the much higher cellular uptake of CPBs** 2** than SPNPs** 2** (Figure 2(b)). The mean fluorescence intensity of CPBs** 2 **in A549 cells is determined to be ~315 versus ~160 for the case of SPNPs** 2**, that is to say, the cellular uptake of CPBs** 2** in A549 cells is almost twice that of SPNPs** 2**. Similar shape-dependent cellular uptake behaviors of CPBs** 2** and SPNPs** 2** were also observed in SH-SY5Y and HUVEC cells by CLSM and flow cytometry (Figures 2(a) and 2(b)). In SH-SY5Y cells, the cellular uptake of CPBs** 2** is proximately twice that of SPNPs** 2**, and in HUVECs, the multiple is about three. The higher cellular uptake of CPBs** 2** over SPNPs** 2** should be caused by the distinct shapes between CPBs** 2** and SPNPs** 2**, since they have the same volume and surface chemistry. Compared to the spherical structure of SPNPs** 2** that possesses small specific surface area, the long and flexible morphological structure of CPBs** 2** may provide significantly larger contact area with cell membranes which results in stronger interaction and adhesion with cell membranes. Such interaction characteristics can kick-start the cellular uptake processes and give rise to higher cellular uptake. Similar explanation has been proposed in the cellular uptake study of carbon nanotubes and carbon spheres [34].

### 2.3. Endocytic Pathway

The endocytic pathways of the FITC-labeled CPBs** 2** and SPNPs** 2** in A549, SH-SY5Y, and HUVEC cells were studied by using several specific endocytic inhibitors: (1) methyl-*β*-cyclodextrin (M*β*CD), an inhibitor to probe caveolae-mediated endocytosis; (2) chlorpromazine, an inhibitor to probe clathrin-mediated endocytosis; (3) cytochalasin B, an inhibitor of micropinocytosis; (4) sodium azide (NaN_3_), an inhibitor of the ATP-dependent endocytosis. The effects of the inhibitors on the cellular uptake were quantitatively evaluated by flow cytometry ([Supplementary-material supplementary-material-1]). Sodium azide has significant inhibitory effects to the CPBs** 2** and SPNPs** 2** uptakes in all the three cell lines indicating that the cellular uptakes are ATP-dependent pathways. Caveolin-mediated endocytosis, clathrin-mediated endocytosis, and macrocytosis are all ATP-dependent processes. It is notable that, for CPBs** 2** and SPNPs** 2**, different cell lines exhibit different endocytic behaviors. In A549 cells, the main endocytic pathway for CPBs** 2** is clathrin-mediated endocytosis, whereas, for the case of SPNPs** 2**, caveolae-mediated endocytosis plays a major role. In SH-SY5Y cells, for both CPBs** 2** and SPNPs** 2**, the main endocytic pathway is clathrin-mediated endocytosis. When compared to the cases of A549 and SH-SY5Y cells, HUVECs, as a type of noncancer cells, exhibit a distinct endocytic pathway. Cytochalasin B shows the most important inhibition in the cellular uptakes of both CPBs** 2** and SPNPs** 2** in HUVECs, whereas M*β*CD has no inhibition in their cellular uptakes, suggesting that the main endocytic pathway for both CPBs** 2** and SPNPs** 2** in HUVECs is micropinocytosis. Notably, in A549 and SH-SY5Y cells, cytochalasin B does not show significant inhibition effect to the cellular uptakes of the samples. From the above in vitro studies, it can be seen that CPBs** 2** have similar endocytic pathways but higher cellular uptakes in A549, SH-SY5Y, and HUVEC cells when compared to SPNPs** 2**.

### 2.4. Permeability in MCs

**Figure 3:**
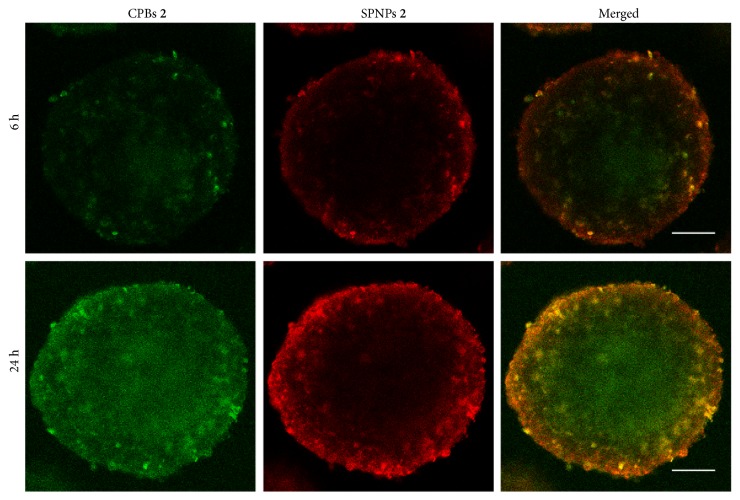
*Permeabilities of CPBs* ***2*** *and SPNPs* ***2*** *in MCs*. CLSM images of the optical slices through the centers of SH-SY5Y MCs coincubated with the FITC-labeled CPBs** 2** and RBITC-labeled SPNPs** 2** together for 6 and 24 h. Scale bars = 50 *μ*m.

When nanomaterials are used as drug carriers for tumor therapy, their permeability in tumor tissues is a crucial factor influencing therapeutic efficiency. The tissue permeabilities of CPBs** 2** and SPNPs** 2** were evaluated by using 3D in vitro tumor models, MCs, prepared from SH-SY5Y cells. To compare their permeabilities accurately and visually, we labeled CPBs** 2** and SPNPs** 2** with FITC and rhodamine B isothiocyanate (RBITC), respectively, and incubated MCs with the labeled CPBs** 2** and SPNPs** 2** in combination for different times, and observed the incubated MCs by CLSM by scanning step by step from the center to the top of the MCs at 20 *μ*m intervals (Figures [Supplementary-material supplementary-material-1] and [Supplementary-material supplementary-material-1]).  Figure 3 shows the typical CLSM images of the optical slices through the center of the MCs after incubation with the labeled CPBs** 2** and SPNPs** 2** together for 6 and 24 h, respectively. Both CPBs** 2** and SPNPs** 2** display a time-dependent penetration process. When the monitoring time was prolonged from 6 h to 24 h, more CPBs** 2** and SPNPs** 2** penetrated to the interior of the MCs, and notably the penetration rate of CPBs** 2** in the MCs is significantly higher than that of SPNPs** 2**. After 6 h incubation, the fluorescence signals from the labeled CPBs (green) can already be seen in the center of the MCs, but the signal from the labeled SPNPs (red) is unobservable there. After 24 h incubation, the signals from the labeled SPNPs are observable in the center of the MCs; in contrast, the signals from the labeled CPBs are much stronger. The higher permeability of CPBs** 2** in MCs than SPNPs** 2** can be reflected vividly by the merged image since the labeled CPBs** 2** and SPNPs** 2** have green and red fluorescence signals, respectively (Figure 3). It can be speculated that the superior permeability of CPBs** 2** in comparison to SPNPs** 2** is attributable to the unique role of shape, since they have the same volume and surface chemistry. The wormlike CPBs** 2** have high flexibility that enable them to fit external environment more easily by adjusting spatial conformation when compared to the spherical SPNPs** 2** with the same volume. This together with their smaller diameter may contribute mainly to the superior permeability of CPBs** 2** in MCs. The high permeability of CPBs was also notified previously by Müllner et al. [35, 36].

### 2.5. In Vivo Blood Circulation Time and in Vitro Phagocytosis by RAW264.7 Cells

The blood circulation time of the intravenously injected nanomaterials affects greatly their passive accumulation in tumors. To measure the blood circulation times of CPBs** 2** and SPNPs** 2**, we labeled the two samples with FITC and injected them into subcutaneous hepatic H22 tumor-bearing mice via tail vein, respectively. The concentrations of CPBs** 2** and SPNPs** 2** in plasma versus time profiles were established, respectively, by recording the fluorescence intensities of plasma samples taken at different time points, and both fit well into one-compartment models ([Supplementary-material supplementary-material-1]). The half-life of SPNPs** 2 **in blood circulation is calculated to be ~6.2 h, which is longer than that of CPBs** 2** (~4.6 h).

Phagocytosis of the monocyte/macrophage system is an important pathway for the clearance of the exogenous substances from the bloodstream [37, 38]. We studied the in vitro macrophage uptakes of the FITC-labeled CPBs** 2** and SPNPs** 2** in RAW264.7 cells by CLSM and flow cytometry (Figures [Supplementary-material supplementary-material-1] A and B). Both CLSM imaging and flow cytometry analyses show that CPBs** 2** are phagocytosed more easily by RAW264.7 cells when compared to SPNPs** 2**. After 4 h incubation, the amount of CPBs** 2** phagocytosed by RAW264.7 cells is almost 2.5 times compared with SPNPs** 2**, which may contribute to the relatively shorter blood circulation time of CPBs** 2** than SPNPs** 2** to a certain extent.

### 2.6. Biodistribution in Tumor-Bearing Mice

**Figure 4:**
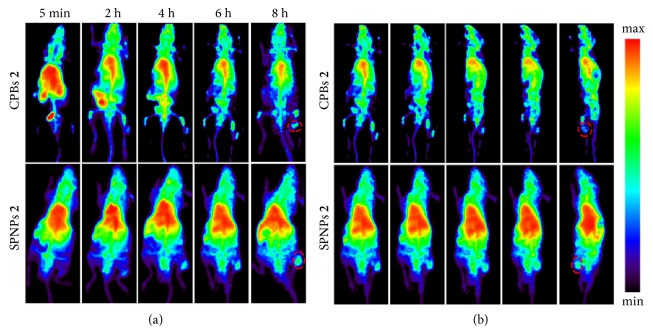
*Biodistributions of CPBs* ***2*** *and SPNPs* ***2***. (a) 3D whole-body microPET images of the subcutaneous H22 tumor-bearing mice at different time points after tail-vein injection of the ^18^F-labeled CPBs** 2** and SPNPs** 2**, respectively. (b) Rotational views of the 3D whole-body microPET images at 6 h after tail-vein injection of the ^18^F-labeled CPBs** 2** and SPNPs** 2**, respectively. The dashed circles indicate the tumor regions. Movie files showing the complete 360° rotational views of the 3D whole-body microPET images of the subcutaneous H22 tumor-bearing mice at different time points after tail-vein injection of the ^18^F-labeled CPBs** 2** and SPNPs** 2** can be found in [Supplementary-material supplementary-material-1] and [Supplementary-material supplementary-material-1], respectively. 3 mice were used for either CPBs** 2** or SPNPs** 2** in the microPET imaging.

The fate of nanomedicines in a living system determines their therapeutic efficiency and side effects. To compare the in vivo behaviors of CPBs** 2** and SPNPs** 2**, we labeled the two polymers with radioactive nuclide fluorine-18 (^18^F) through the reaction between the amino groups in the polymers and* N*-succinimidyl 4-[^18^F]fluorobenzoate ([^18^F]SFB) and traced the biodistributions of the labeled CPBs** 2** and SPNPs** 2** by micropositron emission tomography (microPET) at different time points after tail-vein injection, respectively. This image-based technique is used because it can not only provide precise quantitative biodistribution information in a living body but also reduce the interindividual variability since one animal can be imaged at multiple time points. 3D reconstruction of whole-body microPET images at different time points after tail-vein injection of the radio-labeled samples was conducted (Figure 4; [Supplementary-material supplementary-material-1] and [Supplementary-material supplementary-material-1]), followed by the quantifications of the radioactivities in different tissues. [Supplementary-material supplementary-material-1] shows the biodistribution profiles of the labeled CPBs** 2** and SPNPs** 2** in the heart, liver, kidney, and tumor as well as the remnant percentage of the radioactivity in the whole body at different time points after the injection. It is noteworthy that, for either CPBs** 2** or SPNPs** 2**, the concentration of the injected sample in hearts is the highest among all the test organs over the whole monitoring duration. This is quite different from common nanomaterials, since intravenously injected nanomaterials generally exhibit high liver and spleen uptakes due to the opsonization-induced reticuloendothelial system (RES) capture. The signals acquired in heart region include the signals from blood. The high concentrations of CPBs** 2** and SPNPs** 2** in hearts may be associated with their long retention time in bloodstream due to the high density of PEG chains on their surface. At 5 min postinjection (p.i.), the concentrations in hearts are 16.3% and 16.9% injected dose per gram of tissue (ID/g) for CPBs** 2** and SPNPs** 2**, respectively. Thereafter, their concentrations in hearts decrease gradually as time goes by, and at 8 h p.i., the concentrations are 10.7% and 13.3% ID/g for CPBs** 2** and SPNPs** 2**, respectively ([Supplementary-material supplementary-material-1]A). It can be seen that the concentration of SPNPs** 2** in hearts is always higher than that of CPBs** 2** over the monitoring period, probably because of the longer blood circulation time of SPNPs** 2** than CPBs** 2**. Significant differences in the liver uptake and liver excretion are also observed between CPBs** 2** and SPNPs** 2** ([Supplementary-material supplementary-material-1]B). SPNPs** 2** show higher concentrations in livers than CPBs** 2** at all the test time points except the 5 min point. Within 8 h p.i., the liver uptake of SPNPs** 2** increases from 6.4% to 10.3% ID/g gradually, and the liver uptake of CPBs** 2** decreases from 7.9% to 5.4% ID/g. The significantly different concentrations of CPBs** 2** and SPNPs** 2** in livers can be seen clearly from the microPET images in Figure 4 and [Supplementary-material supplementary-material-1] and [Supplementary-material supplementary-material-1]. In the kidney, at 5 min p.i., the concentration of CPBs** 2** is 9.9% ID/g; in contrast, the concentration of SPNPs** 2** is 5.7% ID/g. At 8 h p.i., the concentrations of CPBs** 2** and SPNPs** 2** in the kidney decrease to 4.2% and 5.5% ID/g, respectively ([Supplementary-material supplementary-material-1]C). The comparisons suggest that CPBs** 2** are much more susceptible to the hepatobiliary and renal excretion than SPNPs** 2**. A rapider body clearance of CPBs** 2** than SPNPs** 2** is also observed ([Supplementary-material supplementary-material-1]D). Notably, in the tumor, their concentrations keep increasing from 5 min to 8 h p.i., and at 8 h p.i., the concentrations of CPBs** 2** and SPNPs** 2** in tumors are 4.5% and 7.2% ID/g, respectively, suggesting that SPNPs** 2** have higher passive tumor targeting ability than CPBs** 2** ([Supplementary-material supplementary-material-1]E).

### 2.7. Extravasation Behaviors from Tumor Vessels

**Figure 5:**
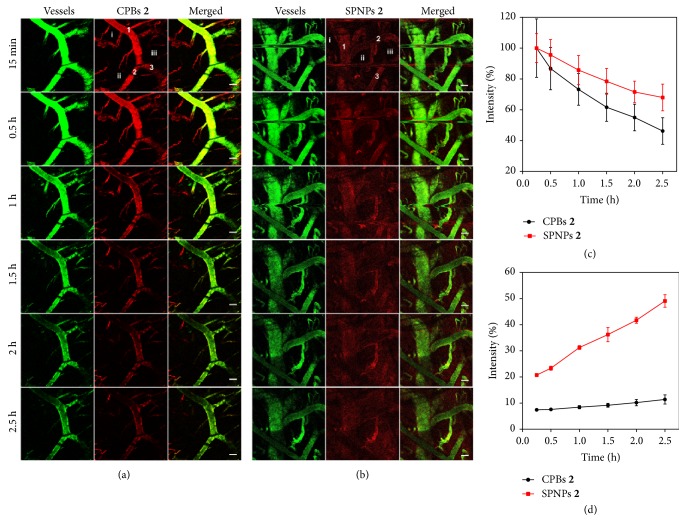
*Real-time observation of in vivo extravasation of CPBs* ***2*** *and SPNPs* ***2*** *from tumor vessels*. Intravital CLSM images of a local tumor area at different time points after tail-vein injection of the RBITC-labeled CPBs** 2** ((a) red) or SPNPs** 2** ((b) red). The blood vessels were stained by FITC-labeled dextran (green). Scale bar = 100 *μ*m. Evolution with time of mean fluorescence intensities of three intravascular areas (c) marked as 1, 2, and 3 in (a) or (b) and three extravascular areas (d) marked as i, ii, and iii in (a) or (b). The mean fluorescence intensities were normalized to the respective intravascular intensities of CPBs** 2** or SPNPs** 2** at 15 min p.i. 3 mice were used for either CPBs** 2** or SPNPs** 2** in the intravital CLSM imaging.

To gain insight into the tumor accumulation of CPBs** 2** and SPNPs** 2**, we monitored their extravasation behaviors from tumor blood vessels by using intravital CLSM after intravenous injection. Figures 5(a) and 5(b) show the real-time CLSM images of a local tumor area at different time points after tail-vein injection of the RBITC-labeled CPBs** 2** or SPNPs** 2**, where blood vessels were stained by FITC-labeled dextran. It can be seen that the signals from the CPBs** 2** in the blood vessels decrease gradually from the beginning of injection, and only very weak signals are observed outside the vessels. In contrast, in the case of SPNPs** 2**, their fluorescence signals in the blood vessels decrease more slowly than those of CPBs** 2** over the monitoring duration. At 0.5 h p.i., obvious signals from SPNPs** 2** can be observed already in the surroundings of the blood vessels, and the signal intensity and distribution area also increase gradually as time goes by. To accurately present the signal evolution with time inside and outside the blood vessels, we quantitatively analyzed the signal intensities of several representative areas, including three intravascular and three extravascular areas (Figures 5(c) and 5(d)). The intravascular and extravascular signal intensities were separately averaged and both were normalized to the intravascular intensity of CPBs** 2** or SPNPs** 2** at 15 min p.i. As shown in Figure 5(c), the intravascular signal intensity of CPBs** 2** decreases more quickly than that of SPNPs** 2**. At 2.5 h p.i., the intravascular signal intensity of CPBs** 2** decreases to ~46.2% of the reference intensity versus ~68.0% for SPNPs** 2**. In contrast, the extravascular signal intensity of CPBs** 2** increases much more slowly than that of SPNPs** 2**. At 15 min p.i., the extravascular signal intensity of CPBs** 2** is ~7.4% of the reference intensity and the percentage for the case of SPNPs** 2** is ~20.7%. At 2.5 h p.i., the extravascular signal intensity of CPBs** 2** increases to ~11.4% of the reference intensity versus ~49.1% for the case of SPNPs** 2**. These results suggest that SPNPs** 2** can extravasate from the tumor vessels more easily than CPBs** 2**. This is not conflictive with the higher permeability of CPBs** 2** in MCs than SPNPs** 2** as discussed above, since the MCs experiments reflect the tissue permeability of CPBs** 2** and SPNPs** 2** under the same concentration, whereas these intravital CLSM experiments reflect their different extravasation ability from tumor vessels. The higher extravasation ability of SPNPs** 2** results in its higher local concentration around the blood vessels compared to CPBs** 2**, which may drive the deeper penetration of SPNPs** 2** into the tumor matrix. The higher extravasation ability of SPNPs** 2** from tumor vessels also explains their higher tumor accumulation demonstrated by the microPET imaging.

## 3. Discussion

The study of the shape effects of nanomaterials on their biological properties demands that the nanomaterials have different shapes but the same volume and surface chemistry. It is indeed a great challenge to fabricate the nanomaterials meeting these conditions. Although polystyrene spheres and rods with the same volume have been prepared in micro- and nanoscales by using film-stretching method [29–33], their hydrophobic nature, limited stability, and lack of reactive groups may greatly limit their applications in biomedical field. Additionally, the film-stretching method requires that the polymer materials have proper T_g_, which is also a limitation for its application.

The high controllability of RAFT and ATRP together with the high efficiency of CuAAC enables us to precisely control the sizes and chemical structures of the CPBs and SPNPs, providing them with the same volume and surface chemistry, which are evidenced by AFM and ^1^H NMR, respectively. The GPC measurements show that the* Ð* values of CPBs** 2** and SPNPs** 2** are 1.13 and 1.11, respectively, demonstrating their narrowly distributed sizes. CPBs** 2** and SPNPs** 2** also have several other advantages, such as desirable stability, high water solubility, and bearing dense PEG chains and abundant reactive groups on surface, which make them very promising for biomedical applications. The significant application potentials of CPBs in biomedical field have also been evidenced by other research groups' work [26, 36, 39–42]. Furthermore, it is reasonable to say that our work provides a practical strategy to the synthesis of nanoscale unimolecular SPNPs with controllable chemical structure and size, whose diameter in dry state can be up to 45 nm. This synthesis strategy may greatly prompt the development of precise synthesis of polymer nanomaterials.

The distinct shapes of CPBs** 2** and SPNPs** 2** plus the same volume and surface chemistry encourage us to investigate the shape effects on their in vitro and in vivo behaviors. As expected, significantly different in vitro and in vivo behaviors were observed between the two materials. Typically, the CPBs had higher cellular uptake in different cell lines, higher phagocytosis by RAW264.7 cells, higher permeability in MCs, and rapider body clearance than the SPNPs, and the SPNPs had longer blood circulation time, higher tumor accumulation and higher ability to extravasate from tumor vessels than the CPBs. Thanks to their good biocompatibility, desirable water solubility, highly controllable structure and size, and abundant reactive groups for functionalizations, both CPBs** 2** and SPNPs** 2 **have great application potentials as delivery carriers for drugs and imaging probes.

## 4. Materials and Methods

### 4.1. Experimental Design

#### 4.1.1. Materials

2,2-Azodiisobutylnitrile (AIBN), glycidyl methacrylate (GMA),* N,N,N*′*,N*′*,N*′′-pentamethyldiethylenetriamine (PMDETA), 4-(*N,N*′-dimethylamino)pyridine (DMAP), 2-bromoisobutyryl bromide, ascorbic acid, cuprous bromide (CuBr), *β*-CD, and propargyl bromide were obtained from J&K Scientific Ltd. PEG monomethyl ether (2 kDa), FITC, and RBITC were purchased from Sigma-Aldrich. PEG** 1** (2 kDa) was supplied by Biomatrik Inc. Trifluoroacetic acid (TFA), sodium azide (NaN_3_), magnesium, ammonium chloride, copper(II) sulfate pentahydrate, and sodium hydride were purchased from Sinopharm Chemical Reagent Co. Ltd., China. Cumyldithiobenzoate [43], 21Br-*β*-CD [44], and [^18^F]SFB [45] were synthesized following published procedures. AIBN was purified by recrystallization from ethanol three times and dried in vacuum at room temperature before used. CuBr was washed with acetic acid and protected in argon atmosphere before used. GMA was passed through a basic alumina column to remove the inhibitor before used.

Nuclear magnetic resonance (NMR) spectra were measured on a Bruker DQX-400 spectrometer with tetramethylsilane as internal standard. The molecular weight and polydispersity index (Ð) of polymers were measured by GPC on a PL-GPC 50 integrated GPC system equipped with a PL aquagel-OH (8 *μ*m, 300 × 7.5 mm) column and an internal refractive index (RI) detector. DMF was used as eluent at 35°C at 1.0 mL/min. AFM measurements were taken on a Vecco multimode V with a Nanoscope Vcontroller (Veeco/Digital instruments, Santa Barbara, CA) operated in the tapping mode using silicon probes (Nanosensors USA, f0=130 kHz) at RT. The AFM samples were prepared by dropping a diluted water solution of CPBs** 2** or SPNPs** 2** (5 × 10^−4^ mg/mL) onto freshly cleaved mica surfaces and dried at room temperature. TEM observations were conducted on a HT-7700 microscope (HITACHI, Japan) operating at an accelerating voltage of 120 kV. The TEM samples were prepared by dropping a diluted water solution of CPBs** 2** or SPNPs** 2** (5 × 10^−4^ mg/mL) onto the surface of carbon support film and dried at room temperature. Fluorescence spectra were measured on a Horiba Jobin Yvon FluoroMax-4 NIR spectrofluorometer at the 480 nm excitation wavelength at room temperature. CLSM images were recorded on a LSM-710 (Zeiss Inc., Germany). Steady-state emission spectra were measured on a Horiba Jobin Yvon FluoroMax-4 NIR spectrofluorometer. The flow cytometry data were recorded on a BD FACASCalibur.

#### 4.1.2. Syntheses and Characterizations

The detailed synthesis procedures and characterizations of the studied polymers can be found in the Supplementary Materials.

#### 4.1.3. Cytotoxicities of CPBs** 2** and SPNPs** 2**

The in vitro cytotoxicities of CPBs** 2** and SPNPs** 2** against the A549, SH-SY5Y, and HUVEC cells were tested by MTT assay. The cells were seeded in a 96-well plate at a density of 5000 cells and incubated with 200 *μ*L of culture medium containing a series of doses of the samples at 37°C for 24 h, respectively. Thereafter, the culture medium in each well was removed and the cells were washed three times with PBS. 20 *μ*L of MTT solution (5 mg/mL) was added to each well and cultured for another 4 h. The supernatant was discarded and then 100 *μ*L of DMSO was added to each well. The values of the plate were recorded by a microplate reader at 570 nm (Safire, Tecan). The results were expressed as the viable percentage of cells after various treatments relative to the control cells without any treatment. Cell viability was calculated by the following formula:

(1) Cell  viability  %=Absorbance test cellsAbsorbance reference cells×100%

#### 4.1.4. Cellular Uptakes of FITC-Labeled CPBs** 2** and SPNPs** 2**

The A549, SH-SY5Y, and HUVEC cells were used to study the cellular uptakes of the FITC-labeled CPBs** 2** and SPNPs** 2**. The cells were seeded into a 6-well plate with a cover glass at a density of 1 × 10^5^ cells and cultured in a humidified atmosphere of 5% CO_2_ in a Dulbecco's modified Eagle's medium (DMEM, Gibco) at 37°C for 24 h to adhere. Then the same amount of FITC-labeled CPBs** 2** and SPNPs** 2** was added to the well, respectively. After another 4 h incubation at 37°C, the coverslips were washed three times with phosphate buffer saline (PBS) to remove the free FITC-labeled CPBs** 2** and SPNPs** 2** in the medium. Thereafter, the cells were fixed and imaged by a CLSM.

To quantitatively study the cellular uptakes of the FITC-labeled CPBs** 2** and SPNPs** 2**, A549, SH-SY5Y, and HUVEC cells were, respectively, seeded into a 12-well plate at a density of 1 × 10^5^ cells and cultured at 37°C for 24 h to adhere. After incubated, respectively, with the same amount of FITC-labeled CPBs** 2** and SPNPs** 2** at 37°C for 4 h and washed with PBS three times, the cells were harvested for flow cytometric analysis.

#### 4.1.5. Endocytic Pathways of FITC-Labeled CPBs** 2** and SPNPs** 2**

A549, SH-SY5Y, and HUVEC cells were, respectively, seeded into a 12-well tissue culture plate at a density of 1 × 10^5^ cells and then allowed to adhere overnight. After preincubated in DMEM medium with M*β*CD (6.5 mg/mL, 1 h), chlorpromazine (10 mg/mL, 1 h), cytochalasin B (10 *μ*g/mL, 1 h), and NaN_3_ (0.5 mg/mL, 1 h), respectively, the cells were further incubated with the FITC-labeled CPBs** 2** or SPNPs** 2** for another 4 h at 37°C. Then the cells were washed three times with PBS, trypsinized, and harvested into 10% FACS buffer (10% fetal bovine serum in PBS). The cells were analyzed by a flow cytometry and 1×10^4^ gated events were recorded. The untreated cells were used for background fluorescence, which was subtracted from test samples. For each sample (CPBs** 2** or SPNPs** 2**), the cells without inhibitor pretreating were taken as control group (set as the 100% uptake efficiency). Three parallel experiments were performed for each sample.

#### 4.1.6. Macrophage Uptakes of FITC-Labeled CPBs** 2** and SPNPs** 2**

RAW264.7 cells were seeded into a 6-well plate with a cover glass at a density of 1 × 10^5^ cells and cultured in a humidified atmosphere of 5% CO_2_ in a DMEM at 37°C for 24 h to adhere. Then the same amount of FITC-labeled CPBs** 2** and SPNPs** 2** were added to the medium, respectively. After another 4 h incubation at 37°C, the cover slips were washed three times with PBS to remove the free FITC-labeled CPBs** 2** and SPNPs** 2** in the medium. Then, the cells were fixed and imaged by a CLSM.

To quantitatively study the cellular uptake, RAW264.7 cells were seeded into a 12-well plate at a density of 1 × 10^5^ cells and cultured at 37°C for 24 h to adhere. After incubated, respectively, with the same amount of FITC-labeled CPBs** 2** and SPNPs** 2** at 37°C for 4 h and washed with PBS three times, the cells were harvested for flow cytometric analysis.

#### 4.1.7. Penetration in MCs

The SH-SY5Y MCs were prepared as described in our previous work [46]. SH-SY5Y MCs with a diameter of 200-300 *μ*m were harvested after approximately 15 days of growth. For each experiment, about 30 spheroids were taken out and transferred to a 10 mL centrifuge tube. Certain amount of FITC-labeled CPBs** 2** and RBITC-labeled SPNPs** 2** were added to the spheroid suspension together and cocultured at 37°C for 6 and 24 h. Thereafter, the medium was removed and the MCs were washed with PBS (pH 7.4) before observed with CLSM. Z-stack images of the treated MCs were obtained by scanning step by step from the center to the top of the MCs at 20 *μ*m intervals.

#### 4.1.8. Blood Circulation Half-Lives of CPBs** 2** and SPNPs** 2**

The hepatic H22 tumor-bearing mice were prepared by subcutaneously injecting 5×10^6^ H22 tumor cells in 100 *μ*L of PBS in the right hind leg of ICR mice (25–28 g). When the tumors reached 150 mm^3^ (7 days after implantation), the mice were divided randomly into two groups (3 mice per group) for CPBs** 2** and SPNPs** 2**, respectively. Thereafter, FITC-labeled CPBs** 2** and SPNPs** 2** in PBS were injected into the mice via tail vein, respectively. Blood samples were collected via eye puncture at different time intervals. Plasma was obtained by centrifuging blood samples at 14000 rpm for 15 min. FITC fluorescence intensity in the plasma was measured by using a fluorescence spectrometer with an excitation wavelength of 480 nm and emission wavelength of 530 nm. The concentrations of the FITC-labeled CPBs** 2** and SPNPs** 2** in the plasma were calculated based on preestablished calibration curves, respectively. The calibration curves were established by adding predetermined amounts of FITC-labeled CPBs** 2** and SPNPs** 2**, respectively, to the plasma samples taken from untreated mice, measuring the fluorescence intensity of the resulting mixture and creating the plots of the fluorescence intensities versus corresponding concentrations of the FITC-labeled samples in plasma.

#### 4.1.9. MicroPET Imaging

The subcutaneous H22 tumor-bearing mouse model was established as stated above. When the tumors reached 150 mm^3^ (7 days after implantation), the mice were divided randomly into two groups (3 mice per group) for CPBs** 2** and SPNPs** 2**, respectively. Thereafter, ^18^F-labeled CPBs** 2** and SPNPs** 2** in PBS were injected into the mice at a radioactive dose of ~200 *μ*Ci per mouse via tail vein under isoflurane anesthesia. MicroPET scans and image analyses were performed on a SNPC-103 microPET scanner. A 1 h dynamic scan was performed immediately after the injection and 10 min static scans were conducted at 2 h, 4 h, 6 h, and 8 h p.i. Image reconstruction was performed after the acquisition and the reconstruction algorithm is OSEM 3D+PSF with the iterations number of 5 times. For each scan, the ROI was plotted over the tumor and major organs using vendor software PMOD on the decay-corrected whole body coronal image. The radioactivity accumulations in different tissues were obtained from mean pixel values within the multiple ROI volume and then converted to MBq per megabyte per minutes using the calibration factor determined for the SNPC-103 PET system. These values were divided by the administrated total radioactivity to obtain (assuming a tissue density to be 1 g/mL) an image-ROI-derived %ID/g.

#### 4.1.10. Real-Time Intravital CLSM Imaging

To establish the experimental tumor model, hepatic H22 tumor cells (5 × 10^6^ cells for per mouse) were planted subcutaneously to ICR mice (25–28 g) at the right axilla. When the tumors reached a proper size, RBITC-labeled CPBs** 2** and SPNPs** 2** were injected into the mice via tail vein at a dosage of 5 mg/kg body weight, respectively (3 mice for either CPBs** 2** or SPNPs** 2**). After anesthetized with isoflurane, the mice were administrated FITC-labeled dextran (100 *μ*L, 1 mg/mL solution in normal saline, the molecular weight of the dextran is about 100 kDa) for vascular staining. Thereafter, the skin on the tumor was cut off immediately and the mouse was fixed on the bench of the CLSM. The tumors were imaged every one minute within 200 minutes.

All animal experiments were implemented according to the National Institute of Health Guide for the Care and Use of Laboratory Animals and approved by the Animal Ethics Committee of Drum Tower Hospital (Nanjing, China).

#### 4.1.11. Statistical Analysis

Quantitative data were expressed as mean ± SD. Statistical comparisons were made by Student's t-test.* P* values less than 0.05 were considered statistically significant.

## Data Availability

All data are available in the manuscript or supplementary materials.

## Supplementary Materials

Supplementary Materials Materials and Methods.* Figure S1*: characterization of 21Br-*β*-CD.* Figure S2*: characterizations of CPBs** 1** and SPNPs** 1**.* Figure S3*: characterizations of CPBs** 2** and SPNPs** 2**.* Figure S4*: AFM analyses of CPBs** 2** and SPNPs** 2**.* Figure S5*: TEM analysis of SPNPs** 2**.* Figure S6*: cytotoxicities of CPBs** 2** and SPNPs** 2**.* Figure S7*: fluorescence properties of the FITC-labeled CPBs** 2** and SPNPs** 2**.* Figure S8*: endocytic pathways of CPBs** 2** and SPNPs** 2**.* Figures S9 and S10*: permeabilities of CPBs** 2** and SPNPs** 2** in MCs.* Figure S11*: blood circulation times of CPBs** 2** and SPNPs** 2**.* Figure S12*: macrophage uptakes of CPBs** 2** and SPNPs** 2**.* Figure S13*: quantitative analyses of the biodistributions of CPBs** 2** and SPNPs** 2**.* Movies S1 and S2*: 360° rotational views of the 3D whole-body microPET images of the subcutaneous hepatic H22 tumor-bearing mice at different time points after tail-vein injection of ^18^F-labeled CPBs** 2** (Movie S1) and SPNPs** 2** (Movie S2), respectively.
