# Supplementary material for: Shape Effects of Cylindrical versus Spherical Unimolecular Polymer Nanomaterials on in Vitro and in Vivo Behaviors
Source: Research (Wash D C). 2019 Apr 24;2019:2391486. doi: 10.34133/2019/2391486 (PMC6750067; doi:10.34133/2019/2391486)
Supplement: Supplementary Materials — Materials and Methods. Figure S1: characterization of 21Br-β-CD. Figure S2: characterizations of CPBs 1 and SPNPs 1. Figure S3: characterizations of CPBs 2 and SPNPs 2. Figure S4: AFM analyses of CPBs 2 and SPNPs 2. Figure S5: TEM analysis of SPNPs 2. Figure S6: cytotoxicities of CPBs 2 and SPNPs 2. Figure S7: fluorescence properties of the FITC-labeled CPBs 2 and SPNPs 2. Figure S8: endocytic pathways of CPBs 2 and SPNPs 2. Figures S9 and S10: permeabilities of CPBs 2 and SPNPs 2 in MCs. Figure S11: blood circulation times of CPBs 2 and SPNPs 2. Figure S12: macrophage uptakes of CPBs 2 and SPNPs 2. Figure S13: quantitative analyses of the biodistributions of CPBs 2 and SPNPs 2. Movies S1 and S2: 360° rotational views of the 3D whole-body microPET images of the subcutaneous hepatic H22 tumor-bearing mice at different time points after tail-vein injection of 18F-labeled CPBs 2 (Movie S1) and SPNPs 2 (Movie S2), respectively. [file 2391486.f1.zip › Supplementary Figures.docx]

**Other supplementary materials for this manuscript include movies S1 and S2.**

**Synthesis of L-**PGMAL-PGMA was synthesized by reversible addition-fragmentation chain transfer (RAFT) mediated radical polymerization with cumyldithiobenzoate as a chain transfer agent. Briefly, glycidyl methacrylate (GMA) (8.5 g, 60 mmol), cumyldithiobenzoate (5.5 mg, 0.02 mmol), anisole (4 mL) and azodiisobutyronitrile (AIBN) (0.5 mg, 0.003 mmol ) were placed in a 25 mL Schlenk flask. The flask was degassed by three freeze-pump-thaw cycles and backfilled with argon. The resulting solution in the flask was stirred at 60 °C for 24 h. The product was isolated and purified by precipitating from tetrahydrofuran (THF) to anhydrous diethyl ether three times and dried under vacuum overnight. The product was obtained as a white powder (2.94 g, 34.6 % conversion rate). ^1^H NMR (400 MHz, CDCl_3_) *δ* (ppm): 0.8-1.2 (3H, -CH_2_-C(C**H_3_**)(C=O)-), 1.8-2.1 (2H, -C**H_2_**-C(CH_3_)(C=O)-), 3.81 (1H, -OC**H_2_**-CH-), 4.32 (1H, -OC**H_2_**-CH-), 3.23 (1H, -OCH_2_-C**H**-), 2.64 (1H, -OCH-C**H_2_**-O), 2.84 (1H, -OCH-C**H_2_**-O).

**Synthesis of S-PGMA**

S-PGMA was synthesized by atom transfer radical polymerization (ATRP) with 21Br-β-CD as a core initiator possessing 21 initiation sites. Briefly, 21Br-β-CD (8.5 mg, 0.002 mmol), GMA (2 g, 0.0141 mol), DMF (2 mL) and ligand *N*,*N*,*N*′,*N*′′,*N*′′-Pentamethyldiethylenetriamine (PMDETA) (15 µL, 0.084 mmol) were placed in a 25 mL Schlenk flask. Then the flask was degassed by three freeze-pump-thaw cycles in liquid nitrogen. After the mixture was frozen again in liquid nitrogen, CuBr (6.3 mg, 0.042 mmol) was added, followed by two freeze-pump-thaw cycles. Thereafter, the flask was sealed under vacuum and the resulting solution in the flask was stirred at 45 ℃ for 10 h. After diluted with THF, the resulting solution was filtered through a column of neutral Al_2_O_3_ to remove copper salts. The product was isolated and purified by precipitating from tetrahydrofuran (THF) to anhydrous diethyl ether three times and dried under vacuum overnight. The product was obtained as a white powder (0.64 g, 32.0 % conversion rate). ^1^H NMR (400 MHz, CDCl_3_) *δ* (ppm): 0.8-1.2 (3H, -CH_2_-C(C**H_3_**)(C=O)-), 1.8-2.1 (2H, -C**H_2_**-C(CH_3_)(C=O)-), 3.81 (1H, -OC**H_2_**-CH-), 4.32 (1H, -OC**H_2_**-CH-), 3.23 (1H, -OCH_2_-C**H**-), 2.64 (1H, -OCH-C**H_2_**-O), 2.84 (1H, -OCH-C**H_2_**-O).

**Synthesis of L-PGA and S-PGA**

To a solution of 0.35 g of L-PGMA (containing ~2.5 mmol epoxy groups) in 15 mL of DMF was added sodium azide (1.3 g, 20 mmol) and ammonium chloride (1.07 g, 20 mmol). The resulting mixture was stirred at 50 °C for 48 h. After the removal of the precipitate by filtration, the product was purified by precipitating from acetone to a saturated NaCl solution 3 times and deionized water twice and dried under vacuum. The product was obtained as a white powder (450 mg). The conversion efficiency of the epoxy group into azido group is demonstrated to be close to 100% by ^1^H NMR. ^1^H NMR (400 MHz, (CD_3_)_2_CO) *δ* (ppm): 0.8-1.2 (3H, -CH_2_-C(C**H_3_**)(C=O)-), 1.8-2.1 (2H, -C**H_2_**-C(CH_3_)(C=O)-), 4.0 (2H, -OC**H_2_**-CH-), 4.1 (1H, -OC**H_2_**-CH-), 3.46 (2H, -CH-C**H_2_**-N_3_).

The S-PGA was synthesized following the same procedures stated above to give the product as a white powder. The conversion efficiency of the epoxy group into azido group is demonstrated to be close to 100% by ^1^H NMR. ^1^H NMR (400 MHz, (CD_3_)_2_CO) *δ* (ppm): 0.8-1.2 (3H, -CH_2_-C(C**H_3_**)(C=O)-), 1.8-2.1 (2H, -C**H_2_**-C(CH_3_)(C=O)-), 4.0 (2H, -OC**H_2_**-CH-), 4.1 (1H, -OC**H_2_**-CH-), 3.46 (2H, -CH-C**H_2_**-N_3_).

**Synthesis of PEG 2**

To a suspension of sodium hydride (0.6 g, 15 mmol) in 20 mL of anhydrous THF was added a solution of PEG monomethyl ether (2 kDa, 6 g, 3 mmol) in 80 mL of anhydrous THF dropwise in an ice water bath and the resulting mixture was stirred at room temperature for 2 h. Thereafter, a solution of propargyl bromide (4.5 g, 30 mmol) in 50 mL of anhydrous THF was added dropwise and the resulting mixture was stirred under reflux for 12 h. After the removal of the precipitate by centrifugation, the product was purified by precipitating from methanol to diethyl ether three times. The product was obtained as a viscous oil (5.9 g) and the reaction efficiency is close to 100% as demonstrated by ^1^H NMR. ^1^H NMR (400 MHz, CDCl_3_) *δ* (ppm): 2.45 (1H, -O-CH_2_C≡C**H**), 3.38 (3H, -O-C**H_3_**), 3.45-3.85 (189H, -OC**H_2_**C**H_2_**-), 4.2 (2H, -O-C**H_2_**C≡CH).

**Syntheses of CPBs 1 and SPNPs 1**

L-PGMA (18.4 mg, containing ~0.1 mmol azido groups), copper(II) sulfate pentahydrate (2.5 mg, 0.01 mmol), PEG **1** (400 mg, 0.2 mmol), PEG **2** (40 mg, 0.02 mmol) and ligand PMDETA (20 µL, 0.1 mmol) were dissolved in 4 mL of DMF in a 25 ml Schlenk flask. The flask was degassed by 2 freeze-pump-thaw cycles in liquid nitrogen. After the mixture was frozen again in liquid nitrogen, a solution of ascorbic acid (35 mg, 0.2 mmol) in 1 mL of DMF was added by syringe followed by 3 freeze-pump-thaw cycles. The resulting solution was stirred at 40 °C for 24 h. After the removal of copper salts by passing through a neutral Al_2_O_3_ column and the solvents under reduced pressure, the product was purified by dialysis against deionized water in a 14 kDa MWCO membrane for two days and then lyophilized. 190 mg of product CPBs **1** was obtained as white powder. ^1^H NMR (400 MHz, D_2_O,) *δ* (ppm): 1.34 (9H, -(C=O)-O-(C**H_3_**)_3_, 3.28 (27H, -O-C**H_3_**), 3.38-3.90 (1792H, -O-C**H_2_**-C**H_2_**-O-).

The synthesis of SPNPs **1** was conducted following the same procedures as the CPBs **1**. ^1^H NMR (400 MHz, D_2_O,) *δ* (ppm): 1.34 (9H, -(C=O)-O-(C**H_3_**)_3_, 3.28 (27H, -O-C**H_3_**), 3.38-3.90 (1792H, -O-C**H_2_**-C**H_2_**-O-).

**Synthesis of CPBs 2 and SPNPs 2**

A certain amount of CPBs **1** or SPNPs **1** was dissolved in anhydrous dichloromethane and then an excess of TFA was added to the solution. After stirring at room temperature for two hours, the product was isolated and purified by precipitating from DMF into anhydrous ether three times. After this process, the Boc protective groups on CPBs **1** and SPNPs **1** are stoichiometrically cleaved.

**Labeling of** **CPBs 2 and SPNPs 2 with FITC or RBITC**

CPBs **1** (20 mg, ~containing 0.001 mmol amino groups), trimethylamine (10 µL, 0.072 mmol) and FITC/RBITC (1 mg) were dissolved in 2 mL of DMF. The resulting solution was stirred at room temperature under darkness overnight. After the removal of the solvents under reduced pressure, the crude product was dialyzed against deionized water in a 14 kDa MWCO membrane for two days to remove unreacted fluorescence dye and lyophilized.

**Radiolabeling CPBs 2 and SPNPs 2 with ^18^F**

To a solution of a sample (CPBs **2** or SPNPs **2**) (1 mg) in 50 μL of dry DMF containing 20 μL of triethylamine was added a solution of *N*-succinimidyl 4-[^18^F]fluorobenzoate ([^18^F]SFB) with a specific activity of ~20 GBq (0.1 μmol) in 50 μL of acetonitrile. The resulting mixture was gently agitated at room temperature for 30 min. The ^18^F-labeled sample was purified by precipitating from DMF to diethyl ether 3 times and the purification was confirmed by instant thin layer chromatography (iTLC) with 80% acetonitrile in water as mobile phase and with detection on an Eckert&Ziegler Radio-TLC Imaging Scanner. After dried under vacuum, the product was formulated in normal saline and passed through a 0.22-μm filter into a sterile vial for in vivo experiments.

**Fig. S1. Characterization of 21Br-β-CD.** ^1^H NMR spectrum of 21Br-β-CD in CDCl_3_.

**Fig. S2. Characterizations of CPBs 1 and SPNPs 1.** ^1^H NMR spectra of CPBs **1** (**A**) and SPNPs **1** (**B**) in D_2_O.

**Fig. S3. Characterizations of CPBs 2 and SPNPs 2.** ^1^H NMR spectra of CPBs **2** (**A**) and SPNPs **2** (**B**) in D_2_O.


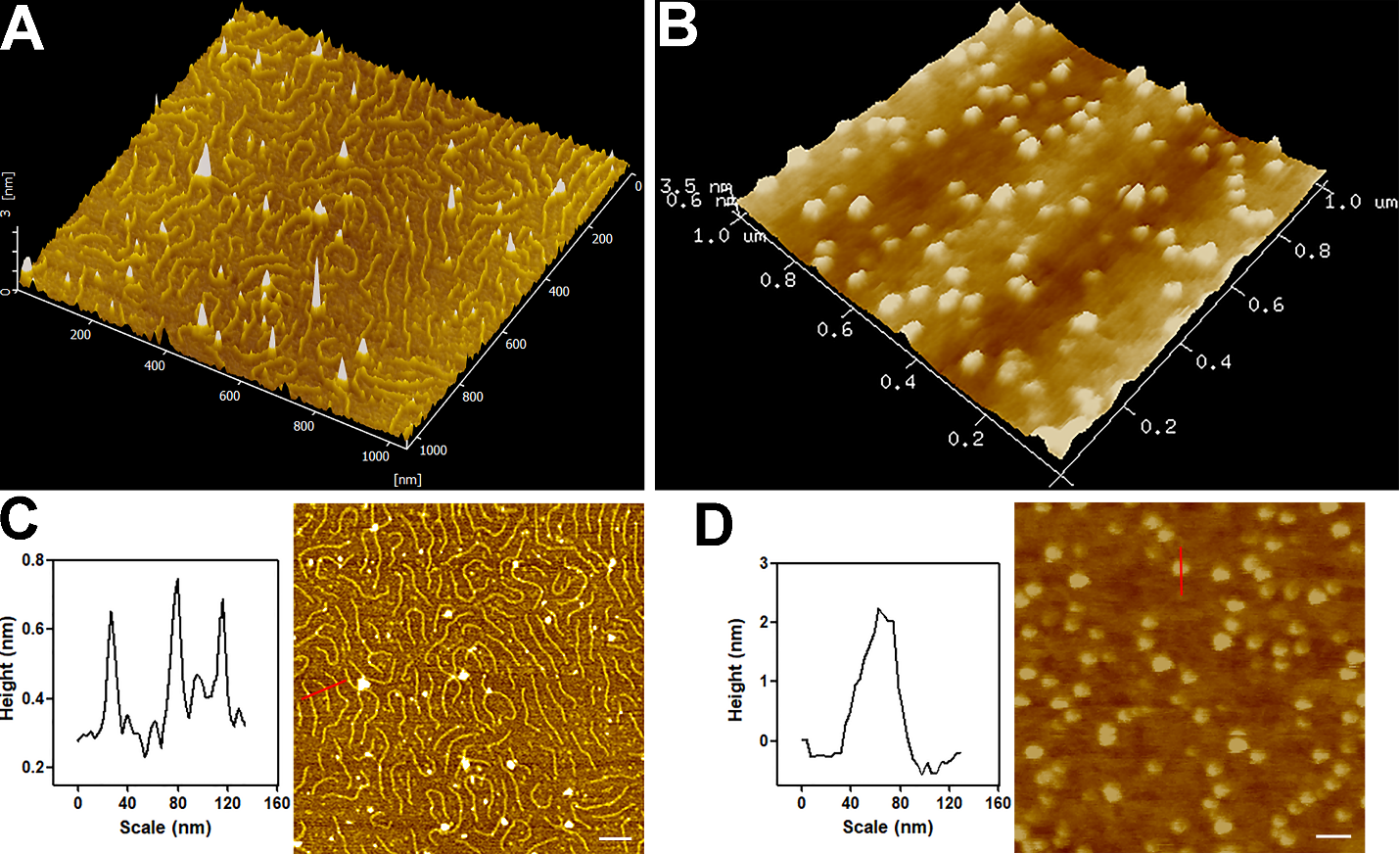


**Fig. S4. AFM analyses of CPBs 2 and SPNPs 2.** 3D AFM images of CPBs **2** (**A**) and SPNPs **2** (**B**), and cross-sections (red line) including AFM height images of CPBs **2** (**C**) and SPNPs **2** (**D**) adsorbed on mica from dilute water solutions. Scale bars = 100 nm.


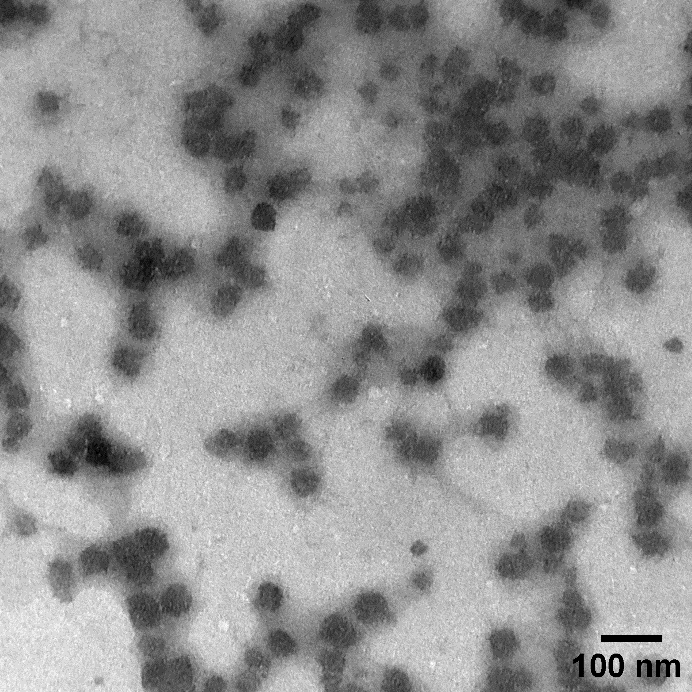


**Fig. S5. TEM analysis of SPNPs 2.** Typical TEM image of SPNPs **2**.

**Fig. S6. Cytotoxicities of CPBs 2 and SPNPs 2.** In vitro cytotoxicities of CPBs **2** (black) and SPNPs **2** (white) against A549 (**A**), SH-SY5Y (**B**) and HUVEC (**C**) cells determined by MTT assay after 24 h incubation.

**Fig. S7. Fluorescence properties of the FITC-labeled CPBs 2 and SPNPs 2.** (**A**) Fluorescence spectra of the water solutions of the FITC-labeled CPBs **2** (black) and SPNPs **2** (red) at a concentration of 200 μg/mL. (**B**) Plots of the fluorescence intensities versus concentrations of the water solutions of the FITC-labeled CPBs **2** (black) and SPNPs **2** (red).


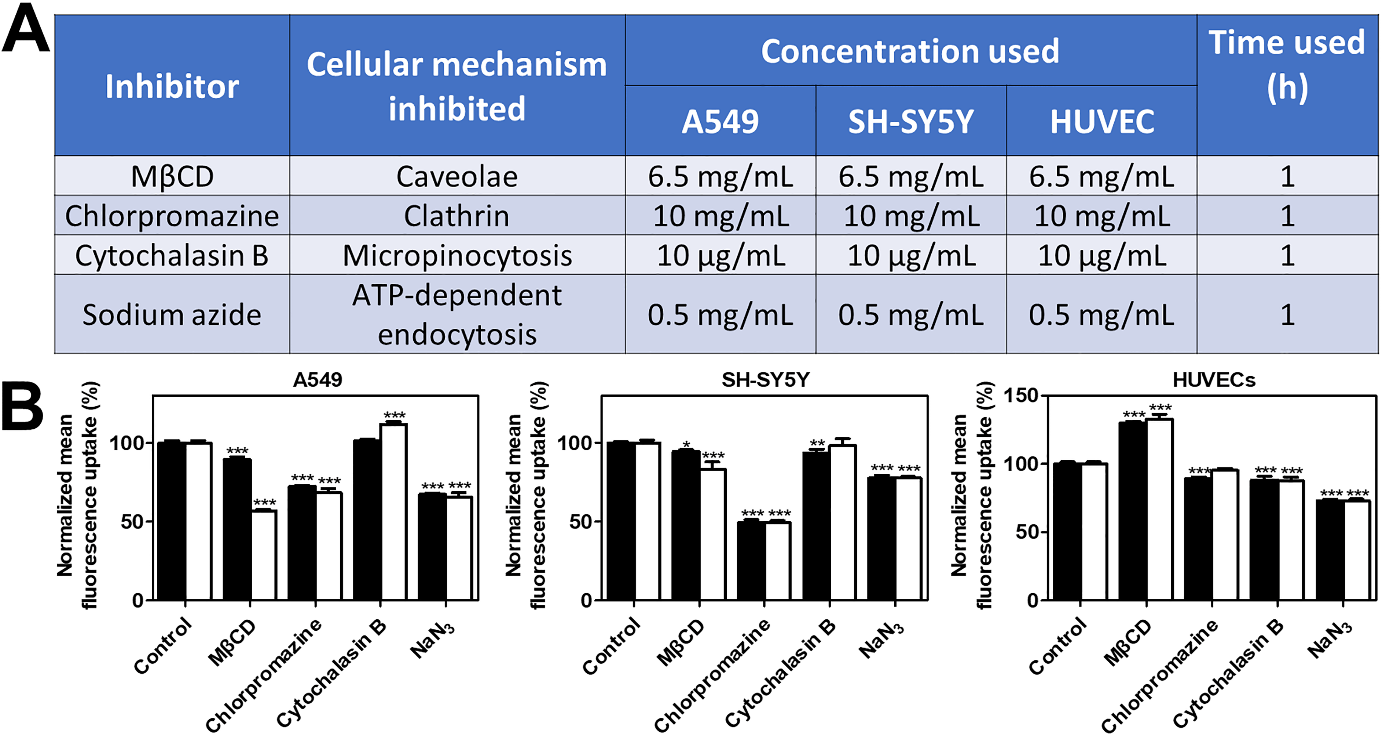


**Fig. S8.** **Endocytic pathways of CPBs 2 and SPNPs 2.** (**A**) The inhibitors, functions, concentrations and time used in the endocytic pathway study. (**B**) Percentages of mean fluorescence uptake in A549, SH-SY5Y and HUVEC cells treated with the specific endocytic inhibitors followed by incubation with the FITC-labeled CPBs **2** (black) and SPNPs **2** (white) determined by flow cytometry. The cells without inhibitor pretreating were taken as control group (set as the 100% uptake efficiency) and the mean fluorescence uptake was normalized to control. Data as mean values ± S.D. (n = 3). **P* < 0.05, ***P* < 0.01, ****P* < 0.001 compared with control.


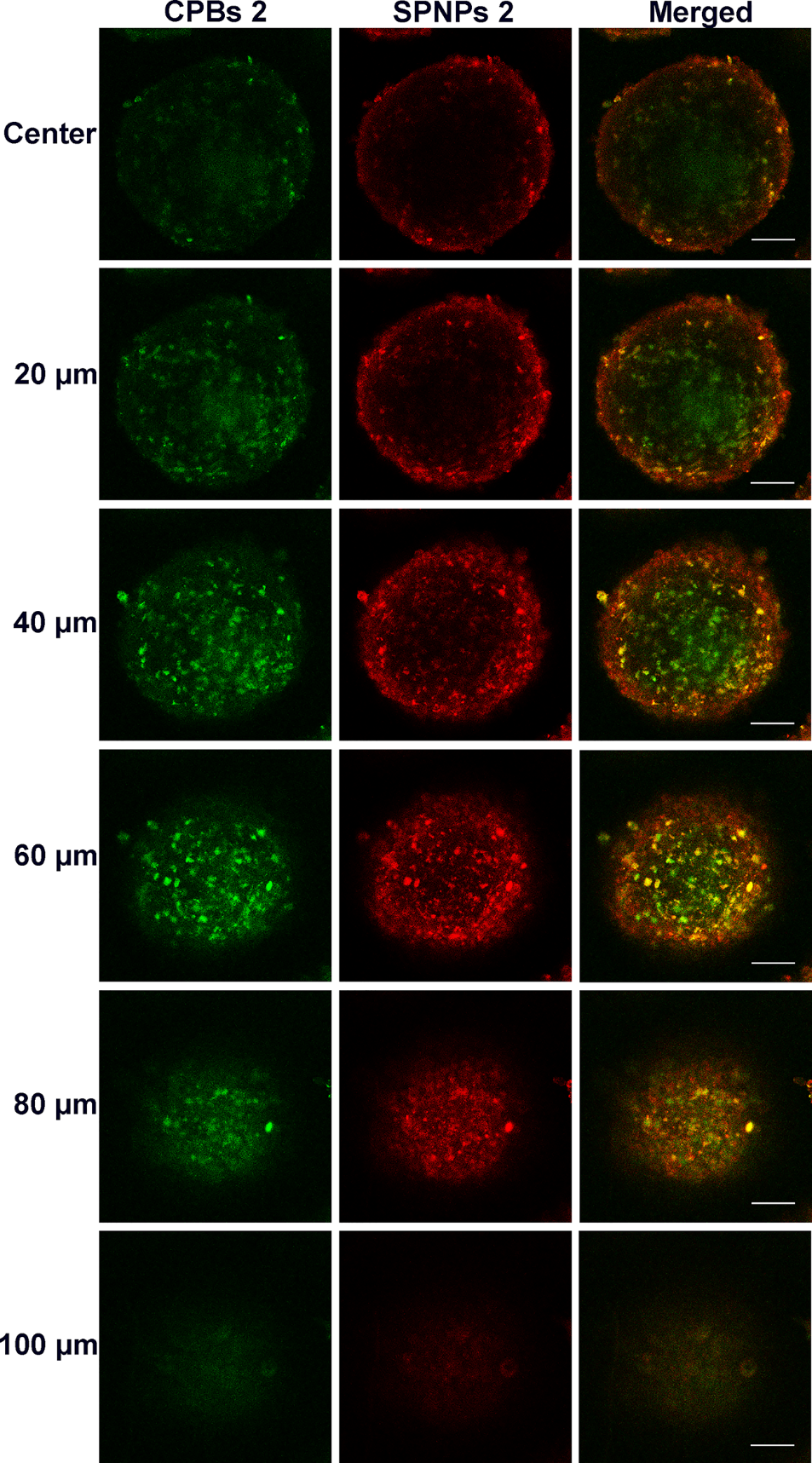


**Fig. S9. Permeabilities of CPBs 2 and SPNPs 2 in MCs.** Typical Z-stack images of SH-SY5Y MCs coincubated with FITC-labeled CPBs **2** and RBITC-labeled SPNPs **2** for 6 h acquired from the center to the top of the spheroid in 20 μm intervals. Scale bars =100 μm.


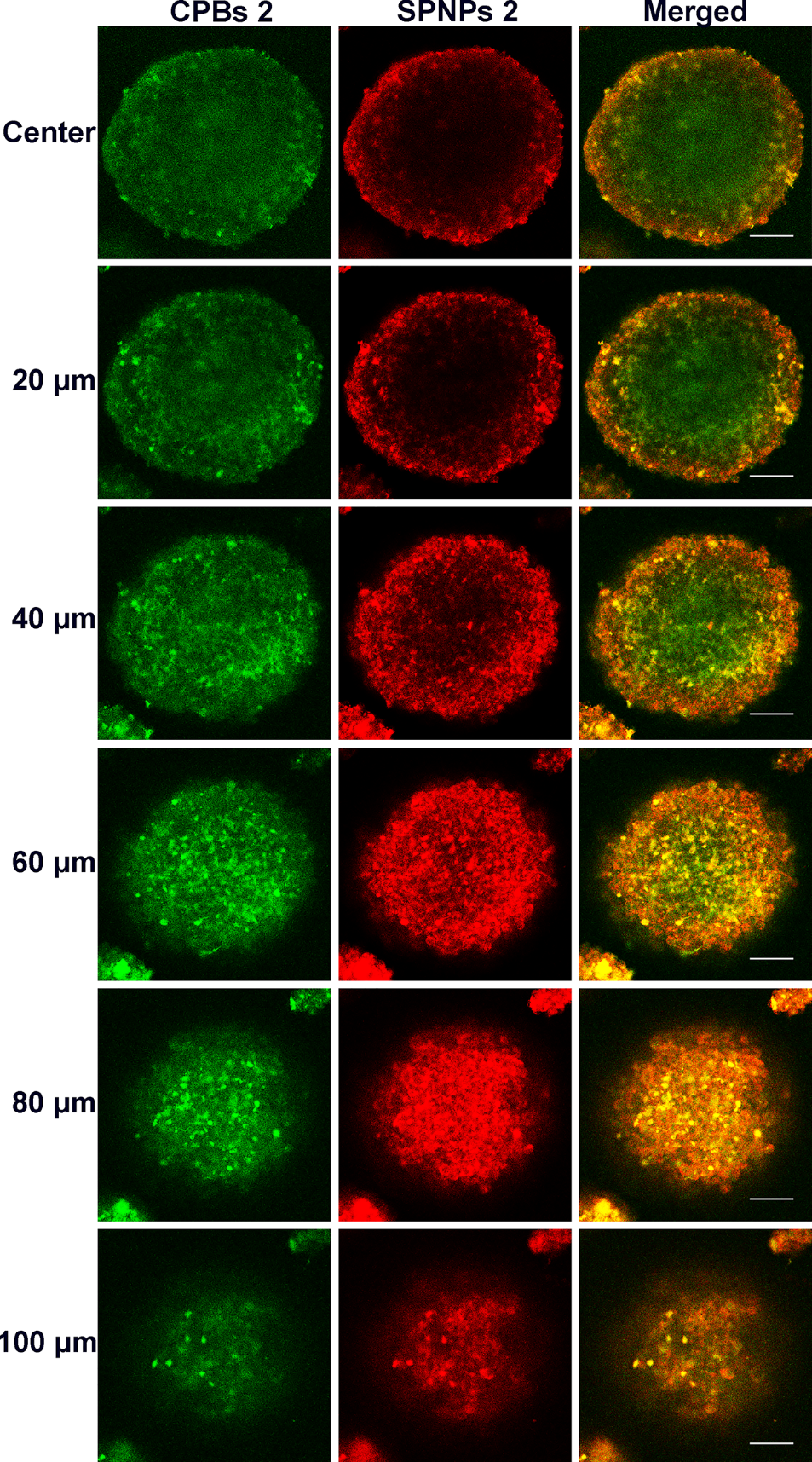


**Fig. S10. Permeabilities of CPBs 2 and SPNPs 2 in MCs.** Typical Z-stack images of SH-SY5Y MCs coincubated with FITC-labeled CPBs **2** and RBITC-labeled SPNPs **2** for 24 h acquired from the center to the top of the spheroid in 20 μm intervals. Scale bars =100 μm.

**Fig. S11*.* Blood circulation times of CPBs 2 and SPNPs 2.** Evolution with time of the concentrations of the FITC-labeled CPBs **2** and SPNPs **2** in the plasma of H22 tumor-bearing mice after tail-vein injection of the FITC-labeled CPBs **2** and SPNPs **2**, respectively.


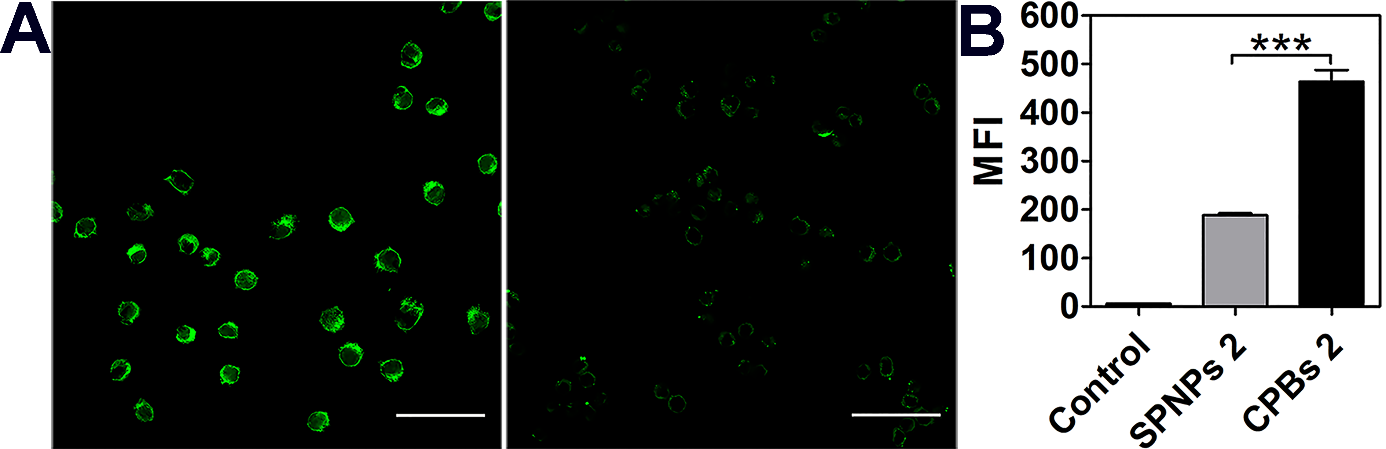


**Fig. S12. Macrophage uptakes of FITC-labeled CPBs 2 and SPNPs 2.** (**A**) CLSM images of RAW264.7 cells after 4 h incubation with the FITC-labeled CPBs **2** (left) and SPNPs **2** (right) at 37 ^o^C, respectively. Scale bars = 20 μm. (**B**) MFI in RAW264.7 cells measured by flow cytometry after 4 h incubation with the FITC-labeled CPBs **2** and SPNPs **2** at 37 ^o^C, respectively. Data as mean values ± S.D. (n = 3). ****P* < 0.001 (CPBs **2** versus SPNPs **2**).

**Fig. S13. Quantitative analyses of the biodistributions of CPBs 2 and SPNPs 2.** Radioactivity quantification in heart (**A**), liver (**B**), kidney (**C**), whole body (**D**) and tumor (**E**) of the H22 tumor-bearing mice at different time points after tail-vein injection of the ^18^F-labeled CPBs **2** (black) and SPNPs **2** (white) determined by image region of interest (ROI) analysis of microPET datasets. The data for the heart, liver, kidney and tumor are expressed as the percentage of ID per gram of tissues and the data for the whole body are expressed as the remnant percentage in the whole body with the radioactivity at 5 min p.i. as 100%. Data as mean values ± S.D. (n = 3).

**Caption for Movies S1 and S2:**

360º rotational views of the 3D whole-body microPET images of the subcutaneous hepatic H22 tumor-bearing mice at different time points after tail-vein injection of ^18^F-labeled CPBs **2** (Movie S1) and SPNPs **2** (Movie S2), respectively.
